# Three newly identified Immediate Early Genes of *Bovine herpesvirus 1* lack the characteristic Octamer binding motif**-** 1

**DOI:** 10.1038/s41598-018-29490-8

**Published:** 2018-07-30

**Authors:** Mayank Pokhriyal, Barkha Ratta, Brijesh Singh Yadav, Ajay Kumar, Meeta Saxena, Om Prakash Verma, Bhaskar Sharma

**Affiliations:** 1 Division of Biochemistry, Indian Veterinary Research Institute, Izatnagar, Bareilly, 243122 UP India; 2 Department of Molecular and Cellular Engineering, Sam Higginbottom University of Agriculture, Technology and Sciences, Allahabad, 211007 UP India; 3 Present Address: IgY Immunologix Pvt. Ltd. H.No. 3-14/2, Survey No. 312, Narsingi Village, Rajendranagar Mandal, Rangareddy District, Hyderabad, 500075 India; 4 Present Address: Bioengineering, University of Information Science & Technology “St. Paul the Apostle”-Ohrid, Patrizanska Str.bb 6000, Ohrid, Macedonia

## Abstract

Only three immediate early genes (IE) BICP0, BICP4 and BICP22 of *Bovine herpesvirus 1* (BoHV-1) are known. These genes are expressed coordinately and their promoters are well characterized. We provide evidence for expression of three additional IE genes of BoHV-1 i.e. UL21, UL33 and UL34. These genes are expressed in the presence of cycloheximide (CH) at the same time as known IE genes. Surprisingly, the promoters of newly identified IE genes (UL21, UL33, UL34) lack the OCT-1 binding site, a considered site of transactivation of the BoHV-1 IE genes. The other difference in the promoters of the newly identified IE genes is the presence of TATA box at near optimal site. However, all the IE genes have similar spatial placements of C/EBPα, DPE and INR elements.

## Introduction

*Bovine herpesvirus 1* (BoHV-1) primarily infects cattle and is associated with respiratory and reproductive venereal diseases along with other clinical symptoms like conjunctivitis, abortion, encephalitis, enteritis, and generalized disease in newborn calves^1^. BoHV-1 is a member of the subfamily *Alphaherpesvirinae* which includes *Human Herpesvirus 1* (HHV-1), the prototype virus for the *Alphaherpesvirinae* subfamily. BoHV-1 has a class D herpesvirus genome. Its genome contains two unique regions, the unique long (UL) and the unique short (US). US region is flanked by inverted repeats (IR) and terminal repeats (TR)^2^. Like HHV-1, viral gene expression in BoHV-1 is regulated temporally in three distinct phases: alpha or immediate-early (IE), beta or early (E) and gamma or late (L)^3–5^. Expression of IE genes initiates from 1 hour post infection (p.i.) and maximizes between 3 to 4 hour p.i^6^.

The IE genes are important for the replication of BoHV-1 as they are required for activation of E and L genes and also for subsequent down regulation of IE genes themselves^5^. During its lytic cycle, BoHV-1 produces three major IE transcripts namely IE4.2 encoding BoHV-1 infective cell protein 4 (BICP4); IE2.9 encoding BICP0 and IE1.7 encoding BICP22^4^. These BICP genes are counterparts to infected cell proteins (ICP) 0, 4 and 22 of HHV-1^7^. The IE transcripts are grouped under two divergent IE transcription unit (IETU); IE4.2 and IE2.9 belongs to IETU1 and IE1.7 comes under IETU2^4,5,8^ but recently it has been shown that BICP0, BICP4 and BICP22 are transcribed from three independent transcription units^9^.

In HHV-1 infected cells, viral gene expression is initiated when the IE genes are transactivated by α gene trans-inducing factor α-TIF, also known as VP16 or Vwm65^10,11^ but α-TIF does not directly bind DNA, rather it forms a complex with the octamer binding protein (OCT-1) which recognizes and binds to the DNA motifs with the consensus sequence TAATGARAT (OCT-1 motif)^10,12^ located in the promoters of the HHV-1 IE genes. This transcriptional program has been well documented in HHV-1, but other members of *Alphaherpesvirinae* seems to follow somewhat similar transcriptional pattern^12–15^. The importance of OCT-1 can be inferred from the fact that it is essential for the sequence specific action of α-TIF^16^.α-TIF provides DNA-binding specificity for formation of the α-TIF-OCT-1 complex because it associates with OCT-1 protein only at the OCT-1-binding motif^17^. The binding of OCT-1 protein with α-TIF brings the complex into the proximity of the transcription start sites^18–21^. Katan *et al*. have shown that deletion in POU domain of OCT-1 protein affects complex formation with α-TIF^22^.

Many α-TIF homologs with similar IE promoter activation properties have been identified in herpes viruses. For example, α-TIF homologs from BoHV-1, varicella-zoster virus, and *Equine herpesvirus 1* transactivates IE promoters and can form α-TIF-OCT-1 containing regulatory complexes on OCT-1 binding motifs^12,17,23,24^. The homolog of tegument protein α-TIF in BoHV-1 is b-transinducing factor (bTIF) and it is responsible for initiating IE viral gene expression in a similar fashion to HHV-1 α-TIF^13,25^.

There is a report which indicates that OCT-1 protein is not the only cellular factor that can transactivate BoHV-1 gene expression^25^. The study suggested the CCAAT enhancer-binding protein alpha (C/EBPα) has the ability to transactivate IE genes. C/EBPs are a family of transcription factors that contains a conserved, basic- leucine zipper domain involved in DNA binding^26^ and belongs to the family of leucine zipper class DNA binding protein^27^.

The transcriptional regulation and computational studies of BoHV-1 in the past have been focused on the three major IE genes^3–5,8^. A random insertional mutagenesis study by Robinson *et al*. mentioned seven genes of unknown regulatory control^28^. This study aimed to ascertain the temporal expression patterns of three BoHV-1 genes viz. UL21, UL33 and UL34 and also evaluated their possible transcriptional regulatory elements.

## Results

### The IE nature of UL21, UL33 and UL34 genes

**Figure 1:**
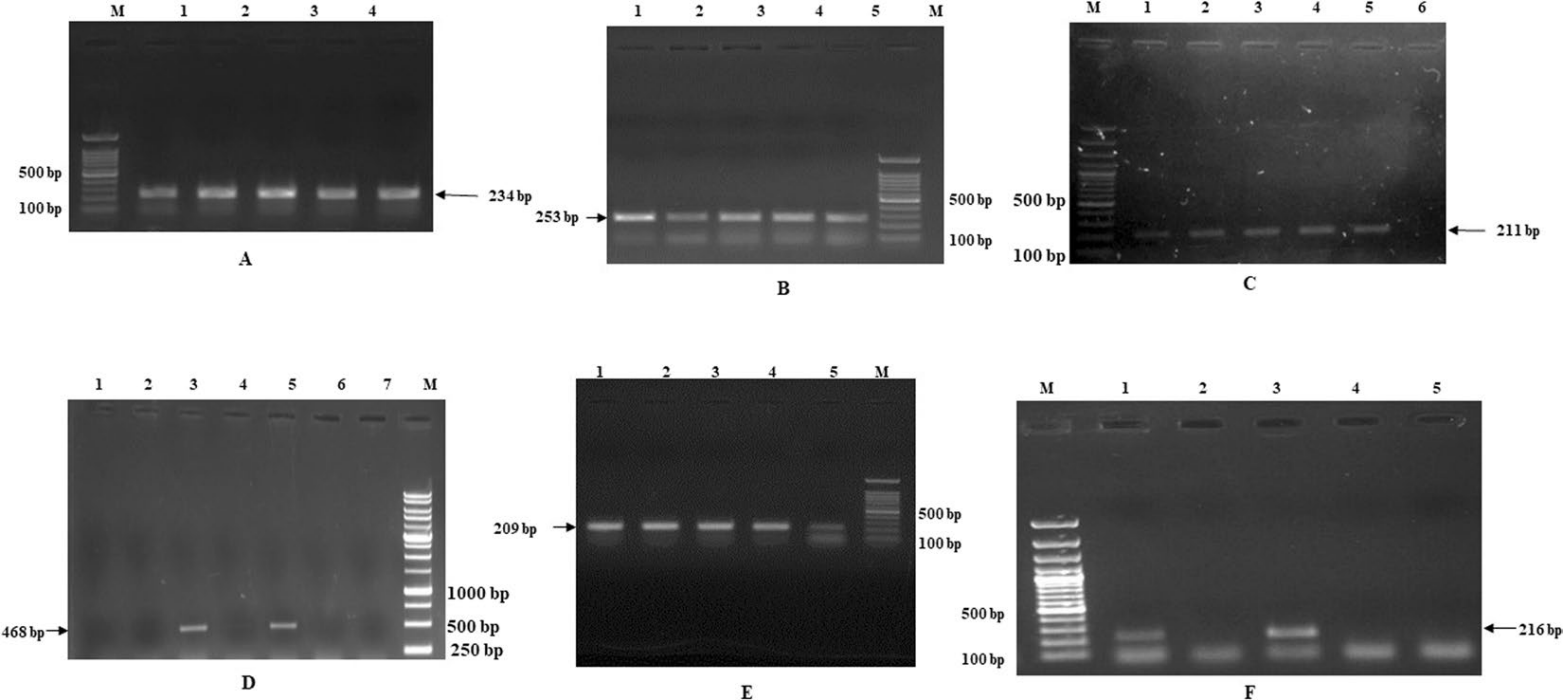
(**A**) Agarose gel Electrophoresis of UL21 PCR amplicon (234 bp) at time points after cycloheximide treatment of BoHV-1 infected MDBK cells. Lane M: 100bp DNA marker (Thermo scientific). Lane 1: 1 hours. Lane 2: 2 hours. Lane 3: 3hours. Lane 4: 4 hours. Lane 5: 5 hours. (**B**) Agarose gel Electrophoresis of UL34 PCR amplicon (253 bp) at time points after Cycloheximide treatment of BoHV-1 infected MDBK cells. Lane 1: 5 hours. Lane 2: 4 hours. Lane 3: 3 hours. Lane 4: 2 hours. Lane 5: 1 hours. Lane M: 100bp DNA marker (Thermo scientific). (**C**) Agarose gel Electrophoresis of UL33 PCR amplicon (211 bp) at time points after Cycloheximide treatment of BoHV-1 infected MDBK cells. Lane M: 100bp DNA marker (Thermo scientific). Lane 1: 1 hours. Lane 2: 2 hours. Lane 3: 3 hours. Lane 4: 4 hours. Lane 5: 5 hours. Lane 6: healthy MDBK used as negative control. (**D**) Agarose gel Electrophoresis of gB PCR amplicon (468 bp) at time points with and without Cycloheximide treatment of BoHV-1 infected MDBK cells in late hours. Lane M: 100bp DNA marker (Thermo scientific). Lane 1: No PCR product of gB gene after 12 hour post infection of BHV-1 without treatment of cycloheximide. Lane 2: No PCR product of gB gene after 12 hour post infection of BHV-1 with treatment of cycloheximide. Lane 3: PCR product of 468 bp of gB gene after 14 hour post infection of BHV-1 without treatment of cycloheximide. Lane 4: No PCR product of gB gene after 14 hour post infection of BHV-1 with treatment of cycloheximide. Lane 5: PCR product of 468bp of gB gene after 16 hour post infection of BHV-1 without treatment of cycloheximide. Lane 6: No PCR product of gB gene after 16 hour post infection of BHV-1 with treatment of cycloheximide. Lane 7: healthy MDBK control. Lane M: 1Kb DNA Ladder (Thermo Scientific). (**E**) Agarose gel Electrophoresis of BICP0 PCR amplicon (209 bp) at time points after Cycloheximide treatment of BoHV-1 infected MDBK cells. Lane 1: 5 hours. Lane 2: 4 hours. Lane 3: 3hours. Lane 4: 2 hours. Lane 5: 1 hours. Lane M: 100bp DNA Ladder (Thermo Scientific). (**F**) Agarose gel Electrophoresis of TK PCR amplicon (216 bp) at time points with and without Cycloheximide treatment of BoHV-1 infected MDBK cells in early hours. Lane M: 100bp DNA marker (Thermo scientific). Lane 1: PCR product of 216 bp of TK gene after 6 hour post infection of BHV-1 without treatment of cycloheximide. Lane 2: No PCR product of TK gene after 6 hour post infection of BHV-1 with treatment of cycloheximide. Lane 3: PCR product of 216 bp of TK gene after 8 hour post infection of BHV-1 without treatment of cycloheximide. Lane 4: No PCR product of TK gene after 8 hour post infection of BHV-1 with treatment of cycloheximide. Lane 5: healthy MDBK control.

To assess if UL21, UL33 and UL34 genes are IE genes, the expression of these genes was studied in BoHV-1 infected cells in the presence and absence of cycloheximide (CH). The transcription of the known IE gene, BICP0 as a control was not inhibited by CH (Fig. 1E) while CH effectively inhibited the expression of early gene thymidine kinase (TK) and late gene glycoprotein B (gB) without affecting the expression of IE genes (Fig. 1D). Figure 1A–C shows the amplification of UL21, UL33, UL34. BICP0, TK and gB genes at different time point in BoHV-1 infected cells in the presence and absence of CH. The sequence of the PCR amplified product was identical to that of the targeted template region (data not shown)^3,6,29^. The GAPDH control confirmed the quantity of the total RNA templates (Supplementary Fig. 1G).

The experiment was repeated three times and found similar results. Results indicate that UL21, UL33 and UL34 are IE genes like BICP0, BICP4 and BICP22 since their expression is not inhibited in presence of CH.

### Promoters of UL21, UL33 and UL34 genes

**Figure 2:**
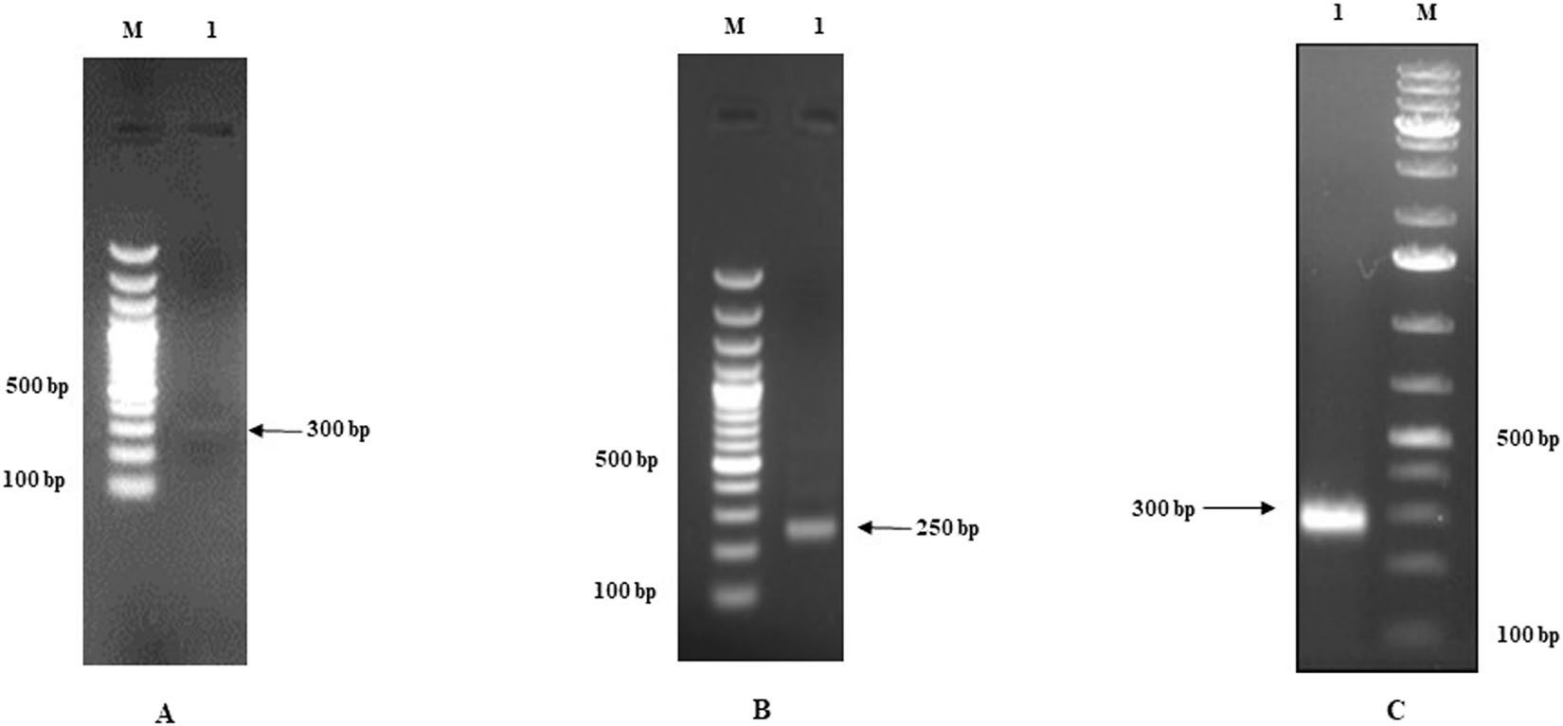
(**A**) Agarose gel Electrophoresis showing RACE amplified products of UL33 gene Lane M: 100bp DNA marker (Thermo scientific). Lane 1: PCR product of 300bp of UL33gene. (**B**) Agarose gel Electrophoresis showing RACE amplified products of UL21 gene. Lane M: 100bp DNA marker (Thermo scientific). Lane 1: PCR product of 250bp of UL21 gene. (**C**) Agarose gel Electrophoresis showing RACE amplified products of UL34 gene Lane M: 100bp DNA marker (Thermo scientific). Lane 1: PCR product of 300bp of UL34 gene.

To delineate the promoter regions of UL21, UL33 and UL34 genes, the transcription start sites (TSS) of these genes were first identified by 5′-RLM-RACE. Each gene gave one amplification product differing in size in RLM-RACE (Fig. 2A–C) suggesting one TSS for each gene. The BICP0, BICP4 and BICP22 also have one transcription start site^9^. The distance between TSS and putative translation start site (ATG) varied among these three genes. This distance was 21 bp in UL21, 127 bp in UL33 and 48 bp in UL34.

### Comparative computational analysis of UL21, UL33 and UL34 genes with BICP0, BICP4 and BICP22

**Figure 3:**
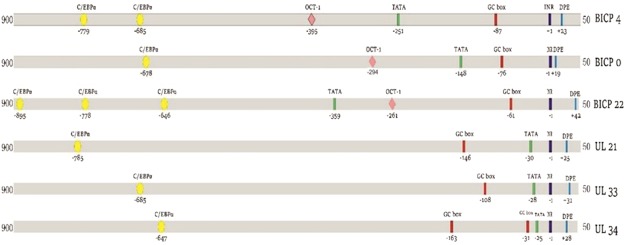
Promoter elements of the promoter regions of UL21, UL33 and UL34.

The comparative computational analysis between the putative promoters of UL21, UL33, UL34 and BICP0, BICP4, BICP22 genes revealed some interesting findings. For computational analysis, we took 1000 bp upstream and 100 bp downstream of the identified TSS sites. The UL21, UL33 and UL34 group showed presence of TATA boxes at optimal position^30–32^ (−30, −28 and −25 bp respectively) from the TSS as shown in Fig. 3 while the TATA box was absent in BICP4, BICP0 and BICP22 at appropriate position though TATA box like sequence was observed at position far upstream from the TSS (−251, −143 and −359 bp respectively). The OCT-1 like sequences were absent in the UL group of IE genes despite intensive examination with relaxed parameters keeping three base mismatch. The OCT-1 motifs were identified in promotor regions of the three known IE genes (Fig. 3). INR (Initiator elements) and DPE (Downstream Processing elements) are important regions in core promoters required for RNA polymerase binding^33–35^. Their presence aids in the prediction of probable promoter sites. INR and DPE were present in both the groups of IE genes and their spatial positioning was almost similar. The optimum position of DPE is at +28 to +32 from the TSS^32,34^. The maximum variation from the optimum position observed was 10 bp.

In BoHV-1, report shows that C/EBPα has the ability to transactivate IE genes^25^. Searching for C/EBPα binding sequences showed conserved C/EBPα motifs in all IE gene (UL21, UL33, UL34, BICP0, BICP4 and BICP22) promoters at similar spatial position relative to the TSS. The nearest conserved position of C/EBPα relative to the TSS was −685, −678, −646, −685, −647, −785 bp in BICP4, BICP0, BICP22, UL21, UL33 and UL34 respectively (Fig. 3).

### Comparative expression of basal promoters of UL 21and BICP0 genes

**Figure 4:**
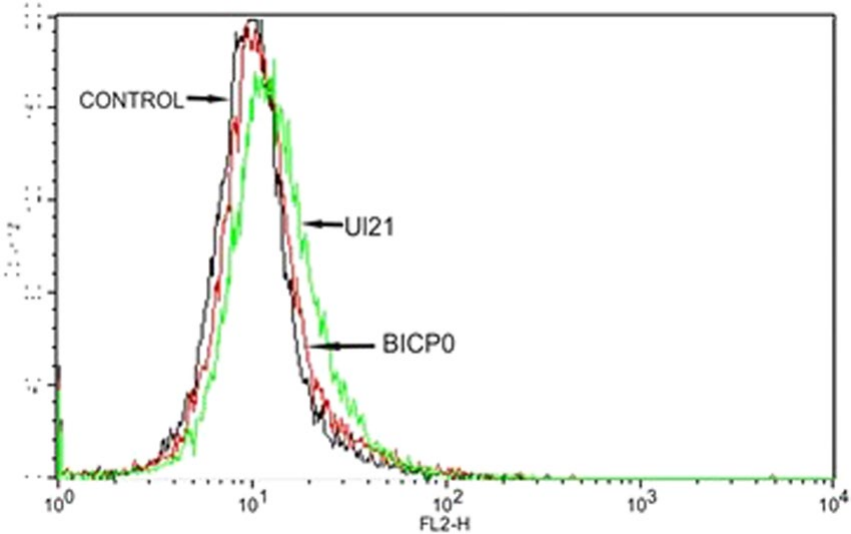
Basal promoter expression by flow cytometry: UL21 shows higher expression compared to BICP0.

**Figure 5:**
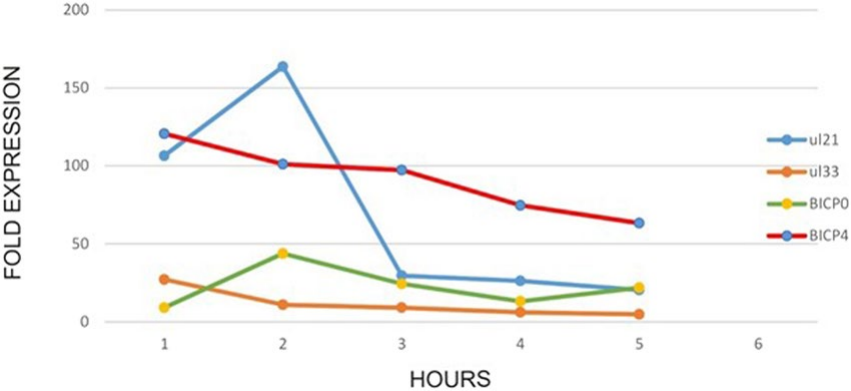
Temporal expression pattern of BICP0, BICP4, UL33 and UL21 genes in Real time PCR.

The UL group of IE genes contains TATA box in optimal positions^30^ while TATA boxes identified in promoter regions of the BICP group were not at the optimal position suggesting that they have no role in RNA polymerase binding. To test if TATA box in optimal position influences basal promoter activity of UL and BICP promoters, the promoter region 300 bp upstream from TSS of one representative from each group (UL21 TATA box at −30 and BICP0 TATA box at −148 base upstream) was cloned in promoterless pDSred Express 2–1 vector and transfected into MDBK cells. The expression of reporter gene was measured in flow cytometer (Fig. 4). The data shows higher expression of UL21 promoter as compared to BICP0 promoter. However, this was not reflected in the temporal expression by real time PCR. Real Time PCR showed almost similar expression of both UL and BICP group of IE genes (Fig. 5). The expression study results suggest that the factors upstream of 300 bp may influence expression *in vivo*. Flow cytometry (translation) and real time PCR (transcription) data reflect two different parameters which are not necessarily comparable. Since, we have used same reporter gene having identical sequence, it is assumed that they will have same degradation, stability, translation efficiency etc. and so may reflect mRNA copy number.

## Discussion

Presence of an OCT-1 motif and its other possible variants is an important feature in all the IE genes in HHV-1 identified to date. It is assumed that binding of OCT-1 by bTIF helps in the recruitment of this protein complex to the OCT-1 motif (IFTATGRAAT sequence motif) to initiate the transcription of IE genes in the BoHV-1 infection process^22^. This is the standard model for the beginning of transcription in HHV-1 and many other herpesviruses as well^4,5,10,12,14,36^. There are reports showing that C/EBPα can transactivate IE genes of BoHV-1^25,37–39^, *in vitro* and also that bTIF can activate promoters that lack OCT-1 motifs provided an alternate mechanism for DNA binding is available^10^. We recently reanalyzed the promoters of known IE gene of BoHV-1^9^ and observed that instead of two^4,5,40^, there are three IE transcription units and each of the transcription units have similarly placed INR, DPE and OCT-1 motifs thus reinforcing the hypothesis of OCT-1 having a role in the transactivation of IE genes in BoHV-1.

In this study, we identified three BoHV-1 IE genes that lack OCT-1 site but have a similarly located C/EBPα site as present in known IE genes. Since C/EBPα has the ability to transactivate genes, it is possible that the newly identified IE genes are getting transactivated through it^25^. This is a reasonable assumption which would need further experimentation to prove it. The second point to consider is the functional role if any for these newly identified IE genes. At present, the expression of these genes in virus infection *in vitro* or *in vivo* is not known consequently no functional role is ascribed to them. Putatively, they code for late proteins (UL21: tegument protein, UL3: DNA packaging, UL34: nuclear egress protein)^28,41^. Based on this information, it is difficult to make any guess on their functional role at present.

Identifying gene expression cascade is important in virus multiplication and infection and can potentially be used for identifying targets for intervention. A new gene expression cascade has been identified in this study. Further work is needed to explore the importance of these IE genes in BoHV-1 and possibly other herpesviruses.

## Methods

### Cell Culture and virus infection

Madin-Darby Bovine Kidney (MDBK) cells were obtained from the National Centre for Cell Sciences (NCCS), Pune, India. The cells were cultured and maintained in DMEM (high glucose) (HyClone) supplemented with 10% FBS (HyClone), penicillin 100 U/mL, streptomycin 100 μg/mL at 37 °C and 5% CO_2_ in 25 cm^2^ -flasks (Nunc). An Indian isolate of BoHV-1 Virus strain (BoHV1/IBR 216 II/ 1976/India) maintained at Immunochemistry laboratory, Division of Biochemistry, IVRI, Izatnagar was used for the study.

### Cycloheximide treatment

Sub-confluent (60–70% confluency) MDBK (maintained at Immunochemistry laboratory, Biochemistry Division of IVRI) monolayer in 12 well culture plate were infected with BoHV-1 at 10 multiplicity of infection (moi) along with CH at a concentration of 100 µg/mL and incubated at 37 °C until harvesting of RNA at different time intervals (1, 2, 3, 4, 5 hr.). CH was used to identify IE transcripts and added as described by Misra *et al*.^3^ and Wirth *et al*.^3,8^. BoHV-1 infected MDBK cells (without CH) were used as control. To check the efficiency of CH treatment, known early and late genes of BoHV-1 were used as controls in PCR. RNA was harvested at 6, 8, 10 and 12, 14, 16 hours post infection for TK for early gene and gB for late gene marker respectively. GAPDH primer pair (GAPDH/F and GAPDH/R as mentioned in Table 1), was used as an internal control for RNA quantity and amplification efficiency.

**Table 1:** Oligonucleotide PCR primer.

Primer Name	Sequence	Position in Genome
UL 33/F	5′ CCCCCCGAGGCGCTGGC 3′	42413–42430
UL33/R	5′ CCCGCCCGAGCCGTGTGC 3′	42229–42246
Ul34/F	5′ CGGCCAGGACGGAAGCAACGAG 3′	41819–41840
Ul34/R	5′ CGCGGGCCTTGATCTTCTCCAGC 3′	41587–41609
Ul21/F	5′ GGGGGCGCGTTTGTGGACTG 3′	68574–68593
Ul21/R	5′ GCCCGAGAGCGCGTTGGTGAT 3′	68359–68380
BICP0/F	5′ GCCTGCATCCGCCGGTGG 3′	102578–102595
BICP0/R	5′ GGCCGCCGTGAGGTCGATGG 3′	102386–102405
BICP4/F	5′ CGGAGAGCAGCGAGGACGACGG 3′	107484–107505
BICP/R	5′ GCTTCGATGGCGGCGGCTATGA 3′	107226–107247
TK/F	5′ GGCGGGCCTGGTTGCGTACTAC 3′	63532 to 63553
TK/R	5′ GGCCGCGAGCATGAGCAGATCT 3′	63727 to 63748
gB/F	5′ CACGGACCTGGTGGACAAGAAG′	624–645
gB/R (kataria *et al*., 1997)	5′ CTACCGTCACGTGCTGTGTAC 3′	1070–1091
GAPDH/F	5′ CAAGGTCATCCATGACAACTTTG 3′	First Strand cDNA Synthesis
GAPDH/R	5′ GTCCACCACCCTGTTGCTGTAG 3′	Kit (#K1612) Thermo Scientific

### RNA Isolation

RNA was extracted at different time intervals i.e., 1, 2, 3, 4 and 5 hour post infection. For extraction of total RNA, 1 mL TRIZOL (Life technologies) was added directly to the virus infected monolayer and mixed by pipetting. The rest of the RNA extraction was as per the manufacturer’s protocols. The RNA pellet recovered after centrifugation at 10,000 × g for 15 minutes was washed once with 70% ethanol and then resuspended in 20 µL of nuclease free water. Before cDNA preparation and PCR amplification, isolated total RNA was treated with DNase I (1µ/µL) (Thermo scientific) at 37 °C for 30 min, followed by enzyme inactivation with 50 mM EDTA (2 µL) at 65 °C for 10 min. Same method for RNA isolation was followed for 6, 8, 10 and 12, 14 and 16 hour post infection, DNase treated RNA was used as negative control in PCR.

### Oligonucleotide primers

BoHV-1 complete genome (AJ004801) from NCBI GenBank was used as reference to design gene specific forward and reverse primers to amplify the 5′ regions of the UL21, UL33 and UL34 genes. For RACE, universal primers (outer and inner) were provided with the RLM-RACE kit to recognize the 5′ end of the cDNA. Two sets of outer and inner (nested) gene specific reverse primers were designed to amplify the part of UL21, UL33 and UL34 genes. All the primer sets for PCR reactions were designed using GeneTool Lite (Biotools Incorporated Ltd.). The primer sequences and other relevant details of all the primers used in this study are given in Tables 1 and 2.

**Table 2:** RACE Primers.

Gene	Inner Primer Sequence	Outer Primer Sequence	Position in Genome
UL 21	5′ CGCCCCCAGCCGCGCAGCTCCAG 3′′	5′ CGCGCCAGGCAGTCCACAAAC 3′	68694–68716 (Inner primer position) 68567–68585 (outer primer sequence)
UL33	5′ TCGAGCTCGCGCGGCGCCACGTC 3′	5′ GCCCGAGCCGTGTGCGATC 3′	42317–42339 (Inner primer position) 42232–42250 (Outer primer position)
UL34	5′ GCGCGCCGGGGGGCCGCGAGAAG 3′	5′ CGCGAAAGGGCCGTGGGTCTA 3′	41696–41708 (Inner primer position) 41560–41580 (Outer primer position)
RACE Primers (Provided in Kit)	5′ CGCGGATCCGAACACTGCGTTTGCTGGCTTTGATG 3′	5′ GCTGATGGCGATGAATGAACACTG 3′	Universal primers provided in the kit First Choice RLM-RACE Kit (Life technologies)

### 5′-RLM-RACE for TSS identification

For RACE, RNA was isolated from BoHV-1 infected MDBK cells in 25 cm^2^ cell culture flask at two hours post infection. RACE was performed using 5′-RLM-RACE (RNA Ligase Mediated Rapid Amplification of cDNA Ends) kit (Ambion, Life Technologies) according to the manufacturer’s recommendations. In brief, 5 µL of cDNA was subjected to the first round of PCR amplification using the adaptor primer (outer) provided in the kit and gene-specific reverse primer. The reaction was carried out in a thermal cycler (Biometra, U.K.). The cycling conditions were denaturation at 95 °C for 2 minutes, followed by 34 cycles of denaturation at 95 °C for 30 seconds, annealing touchdown (from 65 to 55 °C) for 45 seconds and extension at 72 °C for 1 minute, with a final extension at 72 °C for 5 minutes. After amplification, the reaction products were diluted 20-fold with water, and 3 µl DNA were then re-amplified for 30 cycles using the same reaction conditions with nested and adaptor primer (inner primer provided in the kit). Positive PCR products were eluted from 1% agarose gel and ligated into pGEM-TEasy vector (Promega) and sequenced through vendors (Bioserves).

### Reverse transcription and Polymerase Chain Reaction (PCR)

cDNA was synthesized from DNase I treated RNAs using Revert Aid cDNA kit (Thermo Scientific). PCR was performed using the specific primers (Table 1) in 20 µL reaction mix. BICP0 (IE genes) was used as a control to confirm IE genes while TK and gB as a control for and L genes respectively. DNase I treated RNA was used as a control for genomic DNA contamination for each reaction. Each RT-PCR reaction contained the following: 10X Dream Taq buffer, 10pmol forward and reverse primers each, 1 µL DMSO, 1U Dream Taq polymerase (Thermo scientific) and 2 µL of cDNA. The reaction was carried out with an initial denaturation at 95 °C for 10 min followed by 34 cycle of denaturation at 95 °C for 30 Sec, annealing was done at 62 °C for 45 sec followed by extension at 72 °C for 1 min with a final extension at 72 °C for 5 min. The amplified PCR products were cloned and sequenced through vendors (Bioserves) to confirm specific amplification.

### Monitoring the expression of UL21, UL33 and UL34 by real-time PCR

Real time PCR was performed for undefined genes (UL21, UL33 and UL34) along with known IE genes (BICP0, BICP4 and BICP22) with specific primers (Table 1). The data for BICP22 and UL34 were not included because of primer dimers despite several attempts were made to standardize it. Real Time PCR was performed to determine the expression pattern of undefined genes and to compare their expression pattern to known IE genes. Sub-confluent MDBK cells maintained in 12 well plate were infected with BoHV–1 virus at 10 m.o.i. Cells were harvested at different time intervals from 1, 2, 3, 4 and 5 hrs post infection (h.p.i). RNA extraction, DNase I treatment and cDNA synthesis was done as described in previous sections. The reaction was performed by SYBR green method using iCycler iQ™ Real-Time PCR Detection System (BIO-RAD). For each gene, 20 µL reaction mix was prepared in 0.2 ml PCR tube on ice in triplicates along with housekeeping gene Glyceraldehyde 3 phosphate dehydrogenase and non-template controls (NTC). The cycling conditions were 95 °C for 10 min, followed by 40 cycles of 95 °C for 15 sec, 62 °C for 30 sec, 72 °C for 45 sec. The comparative CT (cycle threshold) method was employed for relative quantification of genes.

### Construction of reporter plasmid constructs and Transient Transfection

Basal promoter regions of BICP0 and UL21 were PCR amplified and cloned into promoter less pDSRED Express 2-1 (Clonetech) vector.

All plasmids used for transfection were prepared with Sureprep Plasmid Endofree Maxi Kit (Genetix) according to manufacturer’s instructions. The recombinant plasmids were transfected into 70–80% confluent MDBK cells in a 24 well plate using PolyFect transfection reagent (QIAGEN, Germany) as per the manufacturer’s instructions. A total of 600 ng of recombinant plasmids were mixed with serum free Dulbecco’s Minimal Essential Medium (DMEM, optimum) and 3 µL of Polyfect reagent. All samples were kept in triplicates and incubated for 24 hours.

### Flow Cytometry

Transfected cells were harvested and washed with prewarmed (37 °C) Phosphate Buffer Saline (PBS). Cells were dislodged with 50 µL of prewarmed Trypsin Versene Glucose solution (TVG) and pelleted by adding 300 µL PBS and centrifuged at 3,000 rpm for 5 min. The pellet was resuspended in 300 µL PBS and analyzed in FACScalibur (BD Dickinson, USA) in FL2 channel.

### Computational identification of promoter elements

To analyze the putative promoter regions, 1000 nucleotide upstream and 100 nucleotide downstream of TSS was selected for searching the putative promoter core elements. Promoter regions were identified using the online web server PROMOTER SCAN (http://www-bimas.cit.nih.gov/molbio/proscan/) and the downstream core elements were identified using YAPP online webserver (http://www.gene-regulation.com/cgi-bin/pub/programs/patch/bin/patch.cgi). The promoter sequences were also checked with other softwares like TRANSFAC, BDGP-Neural Network Promoter Prediction.

## Electronic supplementary material

Supplementary Dataset 1
