# Supplementary material for: Three newly identified Immediate Early Genes of Bovine herpesvirus 1 lack the characteristic Octamer binding motif- 1
Source: Sci Rep. 2018 Jul 30;8:11441. doi: 10.1038/s41598-018-29490-8 (PMC6065388; doi:10.1038/s41598-018-29490-8)
Supplement: Supplementary file 1 — Supplementary Dataset 1 [file 41598_2018_29490_MOESM1_ESM.docx]

Three newly identified Immediate Early Genes of *Bovine herpesvirus 1* lack the characteristic Octamer binding motif**-** 1

**Mayank Pokhriyal^1,*^, Barkha Ratta^1^, Brijesh Singh Yadav^1,**^, Ajay Kumar^1^, Meeta Saxena^1^, Om Prakash Verma^***^and Bhaskar Sharma^1, #^**

^1^ Division of Biochemistry, Indian Veterinary Research Institute, Izatnagar, Bareilly – 243122, U.P, India

^*Present address:^ IgY Immunologix Pvt. Ltd. H.No. 3-14/2, Survey No. 312, Narsingi Village, Rajendranagar Mandal, Rangareddy District, Hyderabad- 500075, India

^**Present address^ Assistant professor, Bioengineering, University of Information Science & Technology “St. Paul the Apostle”-Ohrid, Patrizanska Str.bb 6000 Ohrid, Republic of Macedonia

^***^Department of Molecular and Cellular engineering, Sam Higginbottom University of Agriculture, Technology and Sciences, Allahabad – 211007, U.P, India

^1,#^Corresponding Author: [bhaskar@ivri.res.in](mailto:bhaskar@ivri.res.in), bhaskarivri@gmail.com Ph: +91-9457695361

**10 9 8 7 6 5 4 3 2 1 M**


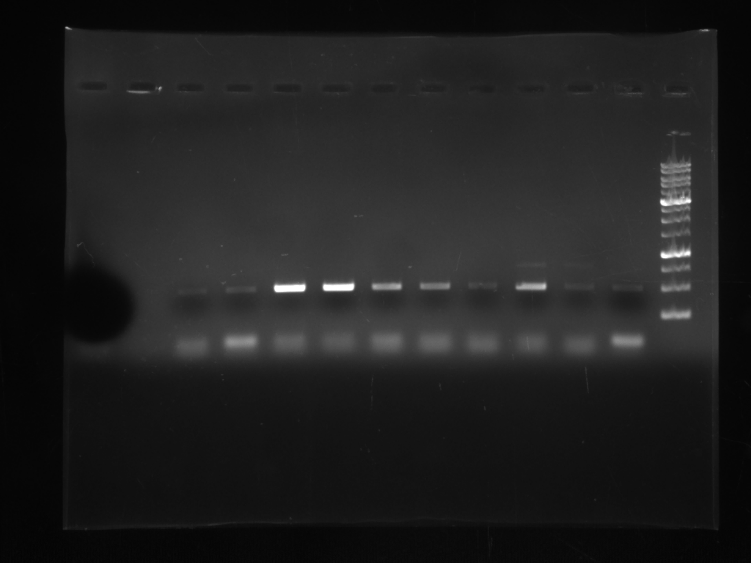


**500 bp**

**250 bp**

**1000 bp**

**496 bp**

**Fig 1G**

**Fig. 1G. Agarose gel Electrophoresis of GAPDH PCR amplicon (496 bp) at time points after Cycloheximide treatment of BoHV-1 infected MDBK cells.**

**Lane M: 1kb DNA marker (Thermo scientific)**

**Lane 1: 1 hours**

**Lane 2: 2 hours**

**Lane 3: 3 hours**

**Lane 4: 4 hours**

**Lane 5: 5 hours**

**Lane 6: 6 hours**

**Lane 7: 8 hours**

**Lane 8: 12 hours**

**Lane 9: 14 hours**

**Lane 10: 16 hours**
